# Supplementary material for: The Potential Role of Wild Suids in African Swine Fever Spread in Asia and the Pacific Region
Source: Viruses. 2022 Dec 24;15(1):61. doi: 10.3390/v15010061 (PMC9867030; doi:10.3390/v15010061)
Supplement: Supplementary file 1 [file viruses-15-00061-s001.zip › viruses-1936302-supplementary.pdf]

Supplementary Materials: The following supporting information can be downloaded at: www.mdpi.com/xxx/s1, Table S1: Definitions of suid populations used in this report; Table S2: Analysis of the type of farming methods used in Asia and the Pacific reported by WOAH members; Table S3: Reports to WAHIS of African swine fever in wildlife in Asia, as of 7th of August 2022; Questionnaire S1.

.

Table S1: Definitions of suid populations used in this report

Suid category	Definition	Subcategory	Definition
Wild[1] suids	Any members of the taxonomic family Suidae that are living freely in the ecosystem without close human management, or that are held in captivity in zoos or breeding programs.<br><br>There are two main types of wild suids, (i) Sus scrofa and (ii) other wild Suidae.	*Sus scrofa*	Feral pigs and wild boar.<br>In general, wild boar are locally endemic and feral pigs have been introduced to an area.
		Other wild suidae	The eleven species of endemic pigs found locally across the Asia and the Pacific region (*Babyrousa celebensis, B. babyrussa, B. togeanensis, Sus barbatus, S. verrucosus, S. celebensis, S. philippensis, S. oliveri, S. ahoenobarbus, S. cebifrons, Porcula salvania)*.
		Hybrids	Wild pigs that are hybrids of wild pig species
Domestic pigs	Any members of the taxonomic family Suidae that are managed by humans, excluding wild pigs held in zoos or breeding programs.<br>Where *Sus scrofa* are farmed, either intensively or extensively, they are referred to as 'domestic' pigs<br>'Domestic pigs' includes farmed wild boar.		

[1] Whilst the term 'wildlife' is used in the WOAH definitions to define feral animals, captive wild animals and wild animals, the term 'wild suids' is used for fluency of language.

Table S2: Analysis of the type of farming methods used in Asia and the Pacific reported by WOAH Members

**Type of domestic pig production in Member**	**Proportion of Members reporting**	**Type of domestic production**	**Number of Members reporting**	**Mean proportion of production across all Members (95% CI)**	**Total number of Members reporting each type of small-scale production**	**Mean proportion of each small-scale production methods across the reporting Members (95% CI)**
Domestic pig production present	72% (18/25)	Large Scale	11	48% (21.7-75.1)	~	~
		Medium scale	14	30% (11.5-47.6)	~	~
		Small scale	15	35% (4.1-65.8)	Free ranging / scavenging (10)	15% (5.81-24.19)
					Semi-intensive (13)	33% (0.3-65.7)

					Intensive (13)<br>Integrated (1)	47.5% (20.4-74.6)<br>N/A
No domestic pig production present	28% (7/25)	~	~	NA	~	~

* Not all Members that specified the type of domestic production system in operation also specified the proportion of the production systems in further detail (percentages). Thus, only those that provided this information were able to be included in this analysis.

Table S3: Reports to WAHIS of African Swine Fever in wildlife in Asia, as of the 7th of August 2022 [105]

Member	Semester	Administrative division	Species	New outbreaks	Susceptible	Cases	Killed and disposed of	Deaths
China (People's Republic of)	Jul-Dec 2018	Heilongjiang	Wild boar		375	77	298	77
	Jan-Jun 2019	Inner Mongolia	Wild boar	1	222	222	12	210
	Jul-Dec 2018	Jilin	Wild boar			1	0	1
	Jul-Dec 2019	Shaanxi	Wild boar	1		9	0	9
	Jan-Jun 2020	Shennongjia	Wild boar	2		7		7
Laos	Jul-Dec 2019	Huaphanh	Suidae (unidentified)	2		6	0	6
Malaysia	Jan-Jun 2021	Beluran	Wild boar	9	21	43	0	43
	Jan-Jun 2022	Bentong	Wild boar	1		1	-	1
	Jan-Jun 2021	Kinabatangan	Wild boar	6	8	10	0	10
	Jan-Jun 2021	Lahad Datu	Wild boar	10	12	16	0	16
	Jan-Jun 2022	Lipis	Wild boar	1		1	-	1
	Jan-Jun 2021	Nabawan	Wild boar	2	1	2		2
	Jan-Jun 2022	Raub	Wild boar	2		3	-	3
	Jan-Jun 2021	Sandakan	Wild boar	4	7	7	0	7
	Jan-Jun 2021	Sandakan	—	2	5	5		5
	Jul-Dec 2021	Semporna	Wild boar	1	1	1	0	1
	Jul-Dec 2021	Semporna	Bearded pig	0		1	0	1
	Jan-Jun 2021	Tawau	Wild boar	4	2	4	0	4
	Jul-Dec 2021	Temerloh	Wild boar	1		12	0	12
	Jul-Dec 2021	Temerloh	Bearded pig	2		12	0	12
	Jan-Jun 2022	Temerloh	Wild boar	1		1	-	1
	Jan-Jun 2021	Tongod	Wild boar	6	7	14	0	14
Korea (Republic of)	Jan-Jun 2022	Boeun	Wild boar	5		67	4	63
	Jan-Jun 2021	Cheorwon	Wild boar	1		1	0	1
	Jan-Jun 2021	Chuncheon	Wild boar	87		87	0	87
	Jul-Dec 2021	Chuncheon	Wild boar	9		31	0	26
	Jan-Jun 2022	Chuncheon	Wild boar	3		21	0	21
	Jan-Jun 2022	Chungiu	Wild boar	4		21	1	20

	Jul-Dec 2021	Danyang	Wild boar	71		71	3	68
	Jan-Jun 2022	Danyang	Wild boar	2		11	0	11
	Jul-Dec 2021	Donghae	Wild boar	1		4	1	3
	Jan-Jun 2022	Donghae	Wild boar	2		4	1	3
	Jan-Jun 2021	Gangneung	Wild boar	72		72	44	28
	Jan-Jun 2022	Gangeung	Wild boar	3		29	15	14
	Jul-Dec 2019	Gangwon-Do	Wild boar			17	2	15
	Jan-Jun 2020	Gangwon-Do	Wild boar	265		269	7	262
	Jul-Dec 2020	Gangwon-Do	Wild boar	182		183	14	169
	Jan-Jun 2021	Gangwon-Do	Wild boar	118		123	4	119
	Jan-Jun 2021	Gapyeong	Wild boar	15		16	0	16
	Jul-Dec 2021	Gapyeong	Wild boar	7		16	1	0
	Jan-Jun 2022	Gapyeong	Wild boar	3		14	0	14
	Jul-Dec 2021	Goseong	Wild boar	7		7	1	6
	Jan-Jun 2022	Goseong	Wild boar	1		1	0	1
Korea (Republic of)	Jul-Dec 2019	Gyeonggi-Do	Wild boar			38	1	37
	Jan-Jun 2020	Gyeonggi-Do	Wild boar	324		332	3	329
	Jul-Dec 2020	Gyeonggi-Do	Wild boar	70		74	10	64
	Jan-Jun 2021	Gyeonggi-Do	Wild boar	54		59	4	55
	Jul-Dec 2021	Hoengseong	Wild boar	44		44		44
	Jan-Jun 2022	Hoengseong	Wild boar	3		12	0	12
	Jan-Jun 2021	Hongcheon	Wild boar	1		1	0	1
	Jul-Dec 2021	Hongcheon	Wild boar	21		45	12	33
	Jan-Jun 2022	Hongcheon	Wild boar	4		15	2	13
	Jan-Jun 2021	Hwacheon	Suidae (unidentified)	2		2	0	2
	Jan-Jun 2021	Hwacheon	Wild boar	57	2	60	0	60
	Jul-Dec 2021	Hwacheon	Wild boar	2		2	1	1
	Jan-Jun 2022	Hwacheon	Wild boar	1		2	0	2
	Jan-Jun 2021	Inje	Suidae (unidentified)	1		1	0	1
	Jan-Jun 2021	Inje	Wild boar	27		27	1	26
	Jul-Dec 2021	Inje	Wild boar	48		68	37	31
	Jan-Jun 2022	Inje	Wild boar	2		3	1	2

	Jul-Dec 2021	Jecheon	Wild boar	44	44	0	44
	Jan-Jun	Jecheon	Wild boar	4	22	0	22
	Jul-Dec 2021	Jeongseon	Wild boar	139	139	11	128
	Jan-Jun 2022	Jeongseon	Wild boar	4	26	0	26
	Jan-Jun 2022	Mungyeong	Wild boar	2	2	1	1
	Jan-Jun 2021	Paju	Wild boar	2	2	0	2
Korea	Jan-Jun 2021	Pocheon	Wild boar	40	41	1	40
(Republic of)	Jan-Jun 2022	Pocheon	Wild boar	1	1	0	1
	Jul-Dec 2021	Pocheon	Wild boar	4	4	2	2
	Jul-Dec 2021	Pyeongchang	Wild boar	15	39	5	34
	Jan-Jun 2022	Pyeongchang	Wild boar	1	1	0	1
	Jul-Dec 2021	Samcheok	Wild boar	1	288	9	19
	Jan-Jun 2022	Samcheok	Wild boar	4	30	5	25
	Jan-Jun 2022	Sangiu	Wild boar	4	36	0	36
	Jul-Dec 2021	Sokcho	Wild boar	1	1	0	1
	Jan-Jun 2022	Uljin	Wild boar	2	10	0	10
	Jul-Dec 2021	Wonju	Wild boar	1	6	0	6
	Jan-Jun 2021	Yanggu	Suidae (unidentified)	1	1	0	1
	Jan-Jun 2021	Yanggu	Wild boar	33	34	0	34
	Jan-Jun 2021	Yanggu	—	1	1	0	1
	Jul-Dec 2021	Yanggu	Wild boar	5	5	1	4
	Jan-Jun 2022	Yanggu	Wild boar	2	2	0	2
	Jan-Jun 2021	Yangyang	Wild boar	16	26	11	15
	Jan-Jun 2022	Yangyang	Wild boar	1	1	0	1
	Jan-Jun 2021	Yeoncheon	Wild boar	66	71	0	71
	Jul-Dec 2021	Yeoncheon	Wild boar	1	2	0	2
	Jul-Dec 2021	Yeongwol	Wild boar	126	126	3	123
	Jan-Jun 2022	Yeongwol	Wild boar	4	50	8	42

Questionnaire S1:

1.1 Respondent details

The data from this survey may be used in research and published. If you would like your data to remain unpublished, please specify ('No') below and your information reported will not be included in any publications. Otherwise, tick 'Yes':

Yes	No

1. Please write your country / territory name:

2. Contact email and name for follow up questions (if you do not require privacy):

3. Please note your organisation

1.2 Wild pigs in your country / territory

4. Please indicate the pig species that are present within your country/territory

⇒ If 'No pigs' are present please end survey here, thank you.

Species	Tick if present:
Wild boar or feral pig *(Sus scrofa)*	
Sulawesi babirusa *(Babyrousa celebensis)*	
Hairy babirusa *(Babyrousa babyrussa)*	
Togian Islands babirusa *(Babyrousa togeanensis)*	
Bearded pig *(Sus barbatus)*	
Javan warty pig, Bawean warty pig *(Sus verrucosus)*	
Sulawesi warty pig *(Sus celebensis)*	
Philippine warty pig *(Sus philippensis)*	
Mindoro (Oliver's) warty pig *(Sus oliveri)*	
Palawan bearded pig *(Sus ahoenobarbus)*	
Visayan warty pig *(Sus cebifrons)*	
Pygmy hog *(Porcula salvania)*	
No pigs	
Other (species not listed – please name)	

5. **For the relevant species present in your country / territory, please fill out the corresponding information:**

⇒ An example row is presented in the table below for *Sus scrofa* (feral pigs) in Australia for reference. Repeat the information for every species that is present.

Species (compete for the species present within the country)	Has ASF been detected in that species?	Is the species protected for conservation?	Are there conservation breeding efforts?	Minimum density of that species (pigs/square km)	Maximum density of that species (pigs/square km)	Approximate % of country where the species is found
**Example row for** Wild boar or feral pig (*Sus scrofa*) **in Australia**	**No**	**No**	**No**	**0.1 pigs/km^2**	**20 pigs/km^2**	**40%**
Wild boar or feral pig (*Sus scrofa*)						
Sulawesi babirusa (*Babyrousa celebensis*)						
Hairy babirusa (*Babyrousa babyrussa)*						
Togian Islands babirusa *(Babyrousa togeanensis)*						
Bearded pig *(Sus barbatus)*						
Javan warty pig, Bawean warty pig *(Sus verrucosus)*						
Sulawesi warty pig *(Sus celebensis)*						
Philippine warty pig *(Sus philippensis)*						
Mindoro (Oliver's) warty pig *(Sus oliveri)*						
Palawan bearded pig *(Sus ahoenobarbus)*						

Visayan warty pig *(Sus cebifrons)*						
Pygmy hog *(Porcula salvania)*						
Other (species not listed – please name and complete the table)						

6. Where are wild *Sus scrofa* or other endemic Suidae found in your country / territory?

Locations wild pigs can be found:	Tick if relevant	Species
Natural areas (e.g. National parks)		
Agricultural areas (e.g. farming/crops/live-stock)		
Semi-rural (e.g. villages)		
Urbanised areas (e.g main towns/city areas)		
**Other instances (specify below):**		

If other instances, please specify below:

1.3 Farming domestic pigs and harvesting wild pigs

7. Are domestic pigs (*Sus scrofa)* farmed within your country / territory? (If 'No', go to Q8).

Yes	No	Unsure

(i) If yes, please indicate the type of farming methods conducted within your country / territory:

Farming method	Tick if relevant	Approximate proportion of national production from each category (%)
Large scale production (>500 sows or >4000 fatteners)		
Medium scale production (5-500 sows or 20-4000 fatteners)		
Small scale production (1-2 sows or 1-20 fatteners)		
⇒ Free range/scavenging (unrestrained)		
⇒ Semi-intensive (confined within large area)		
⇒ Intensive (confined to a pig pen)		
⇒ Integrated (with fish farming)		
⇒ Other (specify below)		

If other methods, please explain in further detail:

8. Do parts of the human population in your country / territory use wild pigs for specific purposes (e.g. hunt for cultural or dietary)? (If 'No', go to Q9).

Y	N	Unsure

(i) If yes, please indicate the purposes for wild pig use:

Wild pig use	Tick if relevant	Relevant species:
Food (diet / consumption)		
Food (ceremonial occasions)		
Hunting/sport		
Feed for farm animals		
Dowries		
Hierarchy ranking		
Rituals (sacrifice, worship, taboo)		
Payment / monetary reasons		
Other (specify below)		

If other purposes, please explain in further detail:

1.4 Transmission of ASF in domestic and wild pigs

9. Has ASF transmitted between wild pigs and domestic pigs in your country / territory? (If 'No', go to Q11).

Y	N	Unsure

(i) If yes, has this transmission been between:

**Sus scrofa**	**Tick if relevant**	
Domestic pigs to wild pigs		
Wild pigs to wild pigs		
Wild pigs to domestic pigs		
Unsure		
**Other wild pigs**		**Species relevant**
Domestic pigs to other wild suidae		
Other wild suidae to other wild suidae		
Other wild suidae to Domestic pigs		
Unsure		

(ii) If transmission is occurring, how it occurring at the wild pig and domestic interface?

**Transmission**	**Tick if relevant**		
	Sus scrofa	**Other wild pigs (specify species to the right if relevant)**	
Direct contact (pig-to-pig)			
Direct contact with dead pig carcasses			
Scavenging of food/waste from domestic pig farms			
Indirect contact (i.e. via human interactions or fomites)			

Spread via effluent from domestic piggery			
Spread via pig products (e.g. pork)			
Vectors (if present)			
Other (specify below):			

If ‘other’ transmission pathways present, please explain in further detail below:

10. Do you have any suggestions on how this transmission could be better controlled?

1.5 Control of ASF in wild pigs.

11. Are there any control or prevention strategies currently being used for ASF in wild pigs in your country / territory? (If 'No', go to Q12).

Y	N	Unsure

(i) If yes, please indicate the control measures for ASF that are used in wild pigs, noting if they are successful:

Control / prevention strategy	Tick if relevant	Species	Successful (Y or N)	If unsuccessful, specify why:
Fencing				
Zoning*				
Biosecurity*				
Surveillance				
Carcass disposal				
Vector control				
Culling/population control				
Border quarantine				
Other (specify below)				
* Zoning: identifying geographical areas/boundaries where specific control strategies (i.e., culling within a certain area) are to be carried out.<br>* Biosecurity: implementation of specific strategies within countries to eliminate/reduce the incursion of the disease (i.e., strict import conditions).				

If other strategies used, please explain in further detail below:

(ii) If culling or population control is conducted, how is this performed and which species are targeted?

Culling method	Y	Species (specify target species)	N
⇒ Shooting on the ground (e.g., hunting)			
⇒ Aerial shooting (e.g., from a helicopter)			
⇒ Trapping			
⇒ Poison baiting			
⇒ Snaring			
⇒ Fertility control			
⇒ Other (please specify):			

(iii) If surveillance is conducted, please indicate the strategies used:

Surveillance strategy	Y	Species (specify target species)	N
⇒ Pig carcass searching			
⇒ Random / frequent testing of wild pig			
⇒ Looking for signs of pig mortality or morbidity			
⇒ Other (please specify):			

12. Do you have a managed hunting system or season for wild pigs within your country / territory? (If 'No', go to Q13).

Yes	No	Unsure

(i) Please indicate the strategies that are relevant:

Pig management strategies	Yes	No	Unsure
A specific hunting period			
Pigs hunted for game			
Pigs hunted for food			
There is a quota/limit when hunting			
There is a target species or demographic of pig hunted (e.g., females)			
Does illegal hunting occur?			
Other (please specify below)			

Please explain in further detail below any additional information on management strategies:

(ii) Please indicate reasons for wild pig management:

Reasons for wild pig management:	Yes	No	Unsure
Prevention of crop damage by wild pigs			
Prevention of potential traffic incidents			
Prevention of invasion to urban areas			
Disease control			
Other (please specify below)			

Please explain in further detail below any additional information on management strategies:

1.6 Maintenance of ASF in wild pigs in your country / territory (if absent, go to Q17)

13. How long has ASF been in wild *Sus scrofa* populations?

⇒ Write the number of months (e.g. 6 means it has been present in wild pigs for 6 months)

14. Do you consider wild *Sus scrofa* to be epidemiologically important in outbreaks in domestic pigs?

Yes	No	Unsure

15. How long has ASF been in other species of wild SUIDAE?

⇒ Write the number of months (e.g. 6 means it has been present in **other species of wild SUIDAE** for 6 months)

16. Do you consider other species of wild SUIDAE to be epidemiologically important in outbreaks in domestic pigs?

Yes	Species	No	Unsure

1.7 Regulations / legislations

17. Do you have regulations or legislation to:

(i) Protect wild pigs for conservation reasons?

Yes (specify below)	No	Unsure

(ii) Control ASF in wild pigs?

Yes (specify below)	No	Unsure

(iii) Regulate hunting in wild pigs?

Yes (specify below)	No	Unsure

18. If relevant, please specify any additional laws or regulations associated with wild pigs and/or domestic pigs for ASF prevention:
